# Herbal formula Xinshuitong capsule exerts its cardioprotective effects via mitochondria in the hypoxia-reoxygenated human cardiomyocytes

**DOI:** 10.1186/s12906-018-2235-4

**Published:** 2018-05-31

**Authors:** Chunjiang Tan, Jianwei Zeng, Yanbin Wu, Jiahui Zhang, Wenlie Chen

**Affiliations:** Fujian Academy of Integrative Medicine, Fujian University of Traditional Chinese Medicine, Fuzhou, Fujian China

**Keywords:** Xinshuitong capsule, Mitochondrial potential, Hypoxia-reoxygenated human cardiomyocytes

## Abstract

**Background:**

The collapse of mitochondrial membrane potential (ΔΨm) resulted in the cell apoptosis and heart failure. Xinshuitong Capsule (XST) could ameliorate left ventricular ejection fraction (LVEF), New York Heart Association (NYHA) classes and the quality of life in patients with chronic heart failure in our clinical study, however, its cardioprotective mechanisms remain unclear.

**Methods:**

Primary human cardiomyocytes were subjected to hypoxia-reoxygenation and treated with XST200, 400 and 600 μg/ml. The model group was free of XST and the control group was cultured in normal conditions. Cell viability, ΔΨm, the activity of mitochondrial respiratory chain complexes, ATPase activity, reactive oxygen species (ROS) and apoptosis cells were determined in all the groups.

**Results:**

The cell viability in the XST-treated groups was significantly higher than that in the model group (*P* < 0.05). Coupled with the restoration of the ΔΨm, the number of polarized cells increased dose dependently in the XST-treated groups. XST also restored the lost activities of mitochondrial respiratory chain complexes I-IV induced by the oxidative stress. The total of mitochondrial ATPase activity was significantly elevated at XST400 and 600 μg/ml compared to the model group (*P* < 0.05). The levels of mitochondrial ROS and the number of apoptosis cells declined in the XST-treated groups compared to those in the model group (*P* < 0.05).

**Conclusions:**

XST, via restoration of ΔΨm and the mitochondrial respiratory chain complexes I-IV activities, and suppression of mitochondrial ROS generation and the apoptosis cells, maintained the integrity of the mitochondrial membrane to exert its cardioprotective effects in the hypoxia-reoxygenated human cardiomyocytes.

## Background

Mitochondria, as the power house of the heart, are highly packed in the cardiomyocytes. Cardiac cells under prolonged hypoxia condition have been shown with the opening of mitochondrial permeability transition pore (MPTP) [1]. Due to the opening of MPTP causes a transient hyperpolarization, followed by depolarization, and subsequently the collapse of the mitochondrial membrane potential (ΔΨm), which is characterized by mitochondrial swelling and uncoupling. Thus, MPTP opening and mitochondrial ΔΨm collapse have been regarded as a primary mediator of apoptosis in the ischemia-reperfusion heart injury [2, 3].

The mitochondrial electron transport chain (ETC) is found in the inner membrane, where it serves as the site of oxidative phosphorylation through the use of ATP synthase. During this chemical process, ROS can be formed as a byproduct of normal cellular aerobic metabolism in the heart [4, 5]. Thus, the major process from which the heart derives sufficient energy can also result in the production of ROS [5]. On the other hand, ROS can depress the activity of mitochondrial ETC and alter ion pump function in heart [6]. Mounting evidence has strongly implicated ROS signaling in the genesis of cardiac hypertrophy [7–9]. Therefore, maintaining the integrity of the mitochondrial membrane, enhancing antioxidant defense may be a therapeutic method for the protection of cardiomyocytes against the injury of ischemia or hypoxia.

Xinshuitong Capsule (XST, awarded the Invention Patent of the People’s Republic of China, No.ZL201210197892.X), a Chinese herbal medicine formula for chronic heart failure (CHF), which consists of Astragali radix, Pseudostellariae radix and Salviae miltiorrhizae radix et al., has the effects of benefiting Qi and Yang, activating blood and eliminating stasis, and inducing diuresis to alleviate edema. Our previous clinical study showed that the CHF patients, who received XST treatment (3 capsules, tid.), were significantly ameliorated in left ventricular ejection fraction (LVEF), New York Heart Association (NYHA) classes, the symptoms and the quality of life compared to the control group [10]. In vitro, XST-treated hypoxia-reoxygenated human cardiomyocytes showed more tolerant to hypoxia stress. The cells exhibited more regular shape and size than the control [11]. However, the drug’s cardioprotective mechanisms remain elusive, especially, its actions on MPTP and mitochondrial apoptosis pathway. Thus, in current experiments, mitochondrial ΔΨm and mitochondrial mass, the activities of the mitochondrial ETC and the mitochondrial ATPase, and their associations with ROS levels and apoptosis cells will be studied in the hypoxia-reoxygenated cardiomyocytes.

## Methods

### Hypoxia-reoxygenated cell model

Primary human cardiomyocyte (HCM) was purchased from American Science Cell Research Laboratories (San Diego. USA). When the cells reached 80–90% confluence, they ere placed on a 96-well plate or a petri dish at a density of 0.75 × 105cells/ml in a hypoxia chamber (80%N_2_,10%H_2_,10%CO_2_ and 0.2%O_2_) for 12 h,11 following by 2 h reoxygenation. During the process of hypoxia and reoxygenation, the study group were exposed to the water-extract of XST (200, 400 and 600 μg/ml, respectively), while the model group was cultured in the identical conditions free of XST treatment, and the control group was cultured in normal conditions. The drug’s low and high concentrations used in the current experiment were comparable to the human serum levels in the previous clinical study.

### Cell viability assay

As described before [12], cell viability was estimated by the assay of 3-[4,5-dimethylthiazol-2-yl]-diphenyl-tetrazolium bromide (MTT, Sigma-Aldrich). Briefly, after treatment, the cells were washed twice with phosphate-buffered saline (PBS, pH 7.4), and then added 100ul MTT in PBS (0.5 mg/ml) and incubated for 4 h at 37 °C. Followed by removing MTT and oscillating for 10 min, cell viability was estimated at absorbance 570 nm by a Tecan Infinite M200 Pro microplate reader (Tecan, Mannedorf, Swizerland).

### Mitochondrial ΔΨm detected by JC-1 staining

As JC-1 (5,5′,6,6′-tetrachloro-1,1′,3,3′-tetraethylbenzimidazolyl-carbocyanine iodide, Beyotime, China) is a lipophilic fluorescent cation that is incorporated into the mitochondrial membrane, where it can form aggregates due to the state of the mitochondrial ΔΨm. This aggregation changes the fluorescence properties of JC-1 from green to orange fluorescence as the ΔΨm increased. After treatment, cells were harvested, re-suspended and incubated with 10 μg/ml JC-1 at 37 °C for 30 min as before [13, 14]. The cells were then washed and centrifuged, the intact living cells stained the mitochondria with JC-1 would exhibit a pronounced orange fluorescence, however, the cells with a breakdown of ΔΨm showed a decrease of the orange fluorescence (or a increase of the green fluorescence). Thus, the intact and injured cells could be distinguished, and the cell populations will be counted according to the different fluorescence by the flow cytometry (BD Biosciences, CA) (JC-1 green: Ex/Em = 485/525 nm; JC-1 red: Ex/Em =535/590 nm).

Similarly, the fluorescence intensity of JC-1 as the index of ΔΨm alterations could be detected by a confocal microscope (Carl Zeiss AG, Oberkochen, Germany), and the ratio of red/green fluorescence intensity is indicated as the alterations of mitochondrial ΔΨm.

For quantification of mitochondrial mass, we used Mitotracker Green probe (Molecular Probes), which preferentially accumulates in mitochondria regardless of the mitochondrial membrane potential and provides an accurate assessment of mitochondrial mass. Firstly, the cells were washed with PBS and incubated at 37 °C for 30 min with 100 nM MitoTracker Green FM (Molecular Probes) and then harvested using trypsin/EDTA and re-suspended in PBS. Fluorescence intensity was detected with excitation and emission wavelengths of 490 and 516 nm, respectively, and values were corrected for total protein (mg/ml).

### Determination of the activities of mitochondrial respiratory chain complexes

According to manufacturer’s instructions, mitochondrial isolation was performed at 4 °C using a Kit for cultured mammal cell (Thermo Scientific Rockford. USA).

The activities of mitochondrial respiratory chain complexes were analyzed by spectrophotometer (Secomam, Domont,France) as described before [13]. Briefly, complex I (NADH dehydrogenase, EC 1.6.5.3) enzyme activity was measured as a decline in absorbance from NADH oxidation by decylubiquinone before and after adding rotenone (St. Louis, MO, USA). Complex II (succinate dehydrogenase, EC 1.3.5.1) activity was determined as a function of the decrease in absorbance from 2, 6-dichloroindophenol reduction. Complex III (ubiquinone cytochrome c oxidoreductase, EC 1.10.2.2) activity was calculated as a function of increase in absorbance from cytochrome c reduction. And complex IV (cytochrome c oxidoreductase, EC 1.9.3.1) activity was measured as a function of the decrease in absorbance from cytochrome c oxidation. Mitochondrial complexes activities were normalized to whole mitochondrial protein content and expressed as arbitrary units.

### Determination of mitochondrial total ATPase activity

Cell mitochondria and submitochondrial particles were prepared as described before [14]. Briefly, the mitochondrial particles were incubated at 37 °C for 60 min in a 0.5 ml medium containing 2 mmol/l ATP, 100 mmol/l NaCl, 20 mmol/l KCL, 5 mmol/l MgCl_2_, 1 mmol/l EDTA in 50 mmol/l Tris–HCl (pH = 7.0). The tubes were chilled immediately and centrifuged at 200×g for 10 min. Inorganic phosphate liberated in the supernatant was calculated as an indication of ATPase activity according to Fiske and Subbarow [15]. Protein determination was carried out in accordance with Lowry [16] with crystalline bovine serum albumin as a standard.

### Determination of mitochondrial ROS

As described before [13], mitochondrial ROS production was determined using Amplex Red (Molecular Probes, Eugene, OR, USA). Briefly, superoxide dismutase (SOD) was added at 40 units/ml to convert all superoxide into H_2_O_2_. Resorufin formation (Amplex Red oxidation by H_2_O_2_) was detected at an excitation/emission wavelength of 545/590 nm using a spectrophotometer (Secomam, Domont, France). Readings of resorufin formation were recorded every 5 min for 30 min, and a slope (i.e., rate of formation) was produced. The slope obtained was converted into the rate of H_2_O_2_ production with a standard curve. The assay was done at 37 °C in 96-well plates using succinate. The data was converted to nmol/mg protein/minute.

### Quantitative assessment of apoptosis cells by flow cytometry

As described before [17], Annexin V-APC/7-AAD Apoptosis Detection Kits (Becton-Dickinson Biosciences) were used to detect apoptosis cells. The cells stained with annexinV+/7-AAD- were considered apoptosis cells, and the percentage of apoptosis cells was determined by flow cytometry.

### Statistical analysis

Software of SPSS Version 19.0 was used for statistical analysis. Numerical data are expressed as means ± SD. The significance of differences was examined using the ANOVA method. Results with *P*<0.05 were considered to be significant.

## Results

### XST increased the viability of hypoxia-reoxygenated HCM

**Fig. 1:**
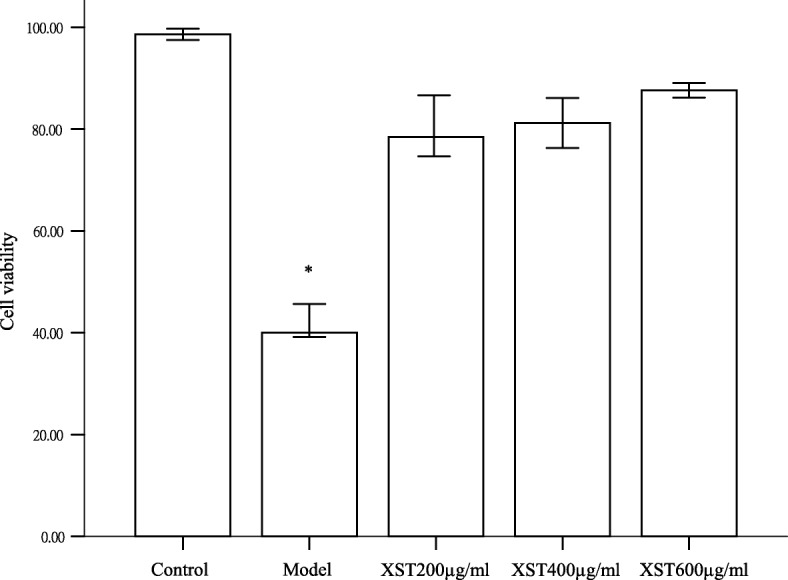
Bar graphs showed the cell viability in the three XST-treated groups were significantly increased compared with the model group. No difference was found between the XST-treated groups or the XST-treated groups compared to the control group. All samples were checked in three independent experiments with three replicates each. Data are represented as the mean ± SD (^*^P<0.05 vs. each of the three XST-treated groups or the control group)

As shown in Fig. 1, the cell viability in the XST-treated 200, 400 and 600 μg/ml group were 77, 81 and 84%, respectively, which showed a significant difference than the model group (42.20%, *P* < 0.05). However, the cell viability exhibited no difference between the three dosages of XST (*P* > 0.05). Under the light microscope, cells in the three XST-treated groups and the control group grew similarly well, and the cells were like in size and shape. By contrast, most of the cells in the model group showed swelling and was out of the regular shape and size (figures not shown). The data indicated that XST could protect the cells against hypoxia-induced injury.

### XST dose-dependently increased the number of polarized cells

**Fig. 2:**
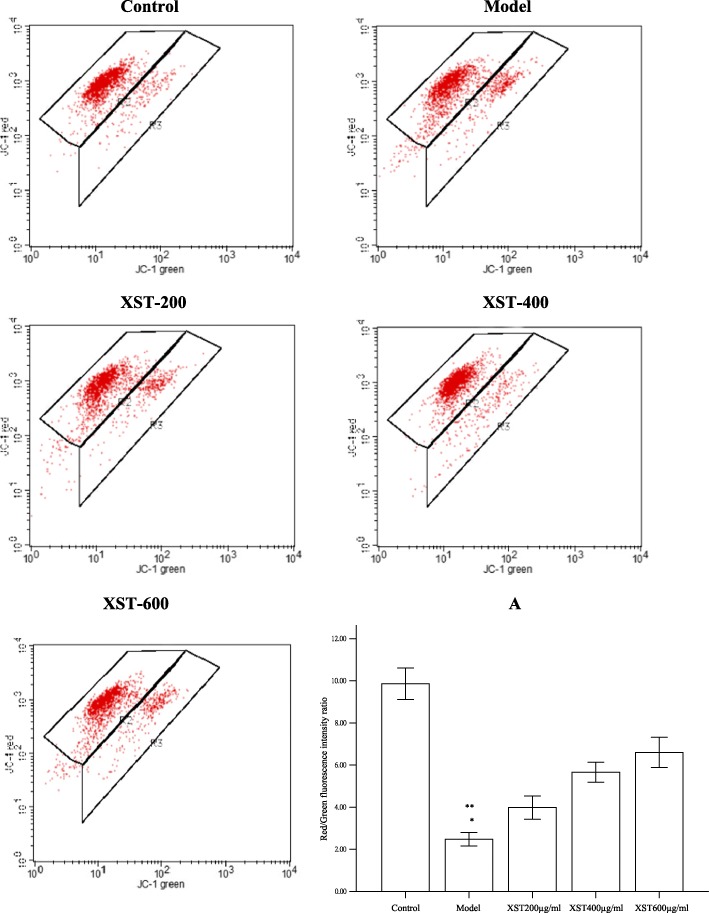
The cell suspensions were analyzed and sorted with flow cytometry according to the different intensity of red and green fluorescence in the five groups. X and Y axes indicated the logarithm of the relative intensity of green and red fluorescence, respectively. Bar graphs. **a** showed the quantitative assessment of the red/green fluorescence intensity ratio. All samples were checked in three independent experiments with three replicates each. Data are represented as the mean ± SD (^*^P<0.05 vs. XST 400 and 600 μg/ml, ^**^P<0.001 vs. the control group)

JC-1 is capable of entering selectively into mitochondria, and the color of the dye changes reversibly from green to orange as the mitochondrial membrane becomes more polarized. Based upon the specific fluorescent characteristics, the cells could be classified into two groups of cells by the flow cytometer, and the two kinds of fluorescence were indicated as the two populations of cells. Quantitative assessment was reflected by the dot plots as indicated in Fig. 2 The ratio of red/green fluorescence, as the index of cell populations, showed a dose-dependent increase in the three XST-treated groups. A significant difference was noted at XST400 and XST600ug/ml compared to the model group (*P* < 0.05), suggesting that XST could increase the polarized cell populations (Fig. 2a).

### XST dose dependently restored the loss of ΔΨm and mitochondrial mass induced by hypoxia

**Fig. 3:**
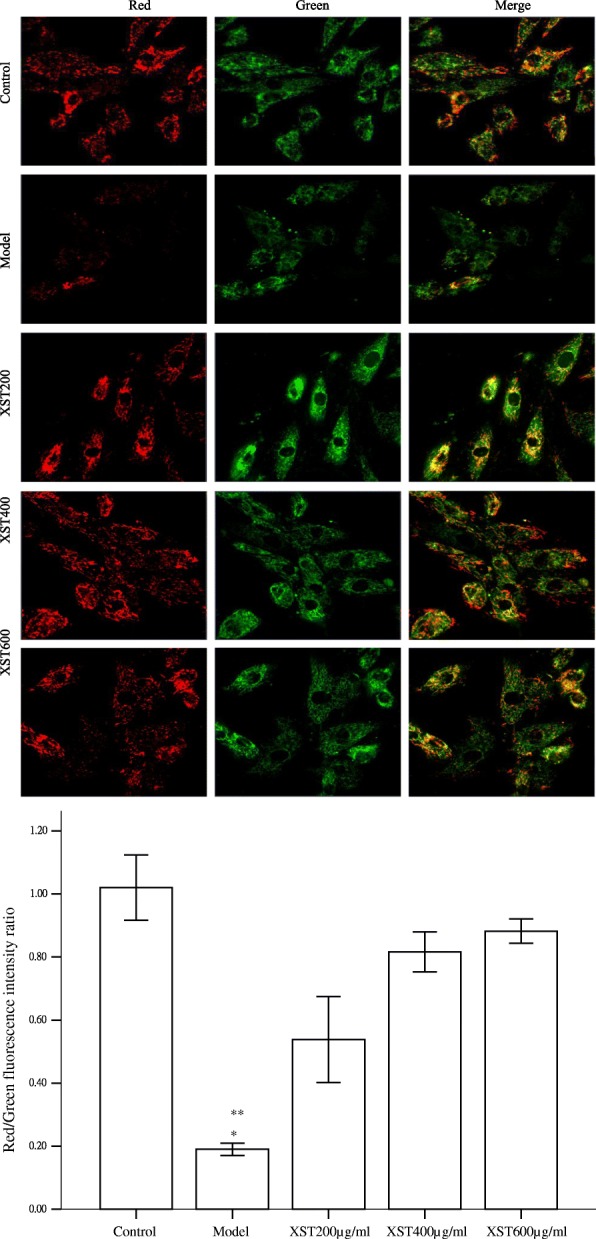
The mitochondrial Δψ_m_ detected by confocal laser scanning microscopy showed that the intensity of red fluorescence of JC-1 aggregates dropped by more than 80% in the model group compared to the control group. XST dose dependent increase in the red fluorescence was noted, indicating that the drug could restore the loss of mitochondrial Δψ_m_ induced by hypoxia. Morphologically, the cells in the XST-treated groups were more regular in size and shape than those in the model group (× 400). Bar graphs showed that the red/green fluorescence intensity ratio increased in a XST dose dependent manner. All samples were checked in three independent experiments with three replicates each. Data are represented as the mean ± SD (^*^P<0.05 vs. XST 200, 400 or 600μg/ml, ^**^P<0.001 vs. the control group)

The intensity of red fluorescence of JC-1 aggregates detected by confocal laser scanning microscopy decreased in the model group, however, XST could dose dependently increase the fluorescence, indicating that the drug could restore the loss of Δψm induced by hypoxia as showed in Fig. 3. Further, the red/green fluorescence ratio in XST-treated 200, 400 and 600μg/ml groups was 55, 81 and 85%, respectively, a significant difference was found in the XST-treated groups compared to the model group (18%). However, no difference was found between XST400, XST600 ug/ml and the control (*P* > 0.05) as indicated by the bar graph Fig. 3a.

**Fig. 4:**
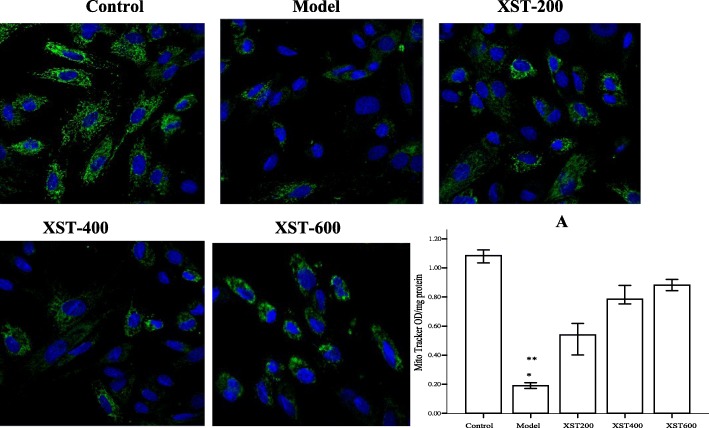
Mitochondrial content (using MitoTracker Green) was detected in the five groups, showing the green fluorescence increased in a XST dose dependent manner. Quantitative assessment showed that XST-treated groups were significantly greater than that in the control (bar graphs (**a**). All samples were checked in three independent experiments with three replicates each. Data are represented as the mean ± SD (^*^P<0.05 vs. XST 400 and 600 μg/ml, ^**^P<0.001 vs. the control group)

Further, an accurate assessment of mitochondrial mass was conducted, showing the green fluorescence increased in the XST-treated groups (Fig. 4). A significant elevation was noted in XST-400 and 600μg/ml groups compared to that in the model group, suggesting the drug prevented the loss of mitochondria in the hypoxia cells. The data was in line with the status of mitochondrial Δψm detected in above experiments. Additionally, morphological observations showed the cells in the XST-treated groups were more uniform in shape and size compared to those in the model group.

### XST restored the mitochondrial electron transport chain complexes activities

**Fig. 5:**
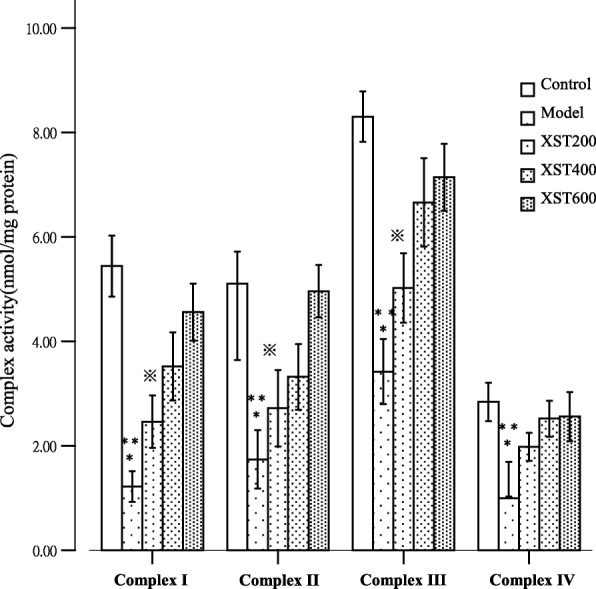
The activities of complexes I-IV were decreased variable in the model group, XST could dose dependently restore the activities of complexes I, II and III, but complex IV showed no difference among the three XST-treated groups. All samples were checked in three independent experiments with three replicates each. Data are represented as the mean ± SD (^*^P < 0.05 vs. XST 200 μg/ml or XST400μg/ml,^**^P < 0.001 vs. the control or XST 600 μg/ml, ^※^*P* < 0.05 vs. XST 400 μg/ml or XST600μg/ml)

The activities of mitochondria complexes I, II, III, and IV were assessed by spectrophotometric methods. As showed in Fig. 5, the activities of complexes I- IV were reduced in varying degrees in the model group. XST could dose dependently restore the activities of complexes I, II and III, but the activity of complex IV showed no difference among XST 200, 400 and 600 μg/ml groups. Complexes I-IV activities were significantly elevated in XST 200 and 400 μg/ml groups than those in the model group (*P* < 0.05); in the XST 600 μg/ml group, the activities of complexes I-IV restored nearly to the normal levels. The data indicated that the drug could dose dependently restore the activities of the mitochondrial electron transport chain complexes I-IV.

### XST increased mitochondrial total ATPase activity in the hypoxia-reoxygenated HCM

**Fig. 6:**
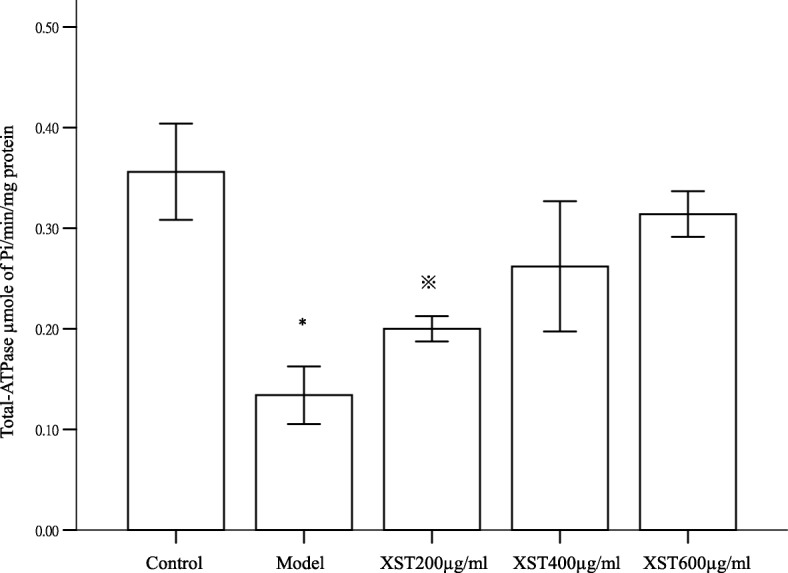
Mitochondrial ATPase activity in cardiomyocytes was determined by estimating the amount of ATP hydrolysis in terms of inorganic phosphorus (Pi) liberated in the cell supernatant. A significant decrease was found in the model group compared to that in the control. Three XST-treated groups exhibited a dose-dependent increase in the activity as showed in bar graphs. All samples were checked in three independent experiments with three replicates each. Data are represented as the mean ± SD (^*^P < 0.05 vs.XST 400 and 600 μg/ml, ^※^*P* < 0.05 vs. 600 μg/ml)

Mitochondrial total ATPase activity was determined by estimating the amount of ATP hydrolyzed in terms of inorganic phosphorus (Pi) liberated in the cell supernatant. As shown in Fig. 6, the ATPase activity in the model group decreased about 70% compared to the control (*P* < 0.05), however, the activity increased about 40, 52 and 60% in the XST-treated 200, 400 and 600 μg/ml groups compared to the model group (*P* < 0.05), which indicated that XST dose dependently increased the mitochondrial ATPase activity induced by hypoxia. The XST-induced elevation of ATPase activity was correlated with the increase in mitochondrial Δψm and the activities of mitochondrial complexes I-IV in the XST-treated groups (Figs. 3 and 5).

### XST decreasedmitochondrial ROS production in the hypoxia-reoxygenated HCM

**Fig. 7:**
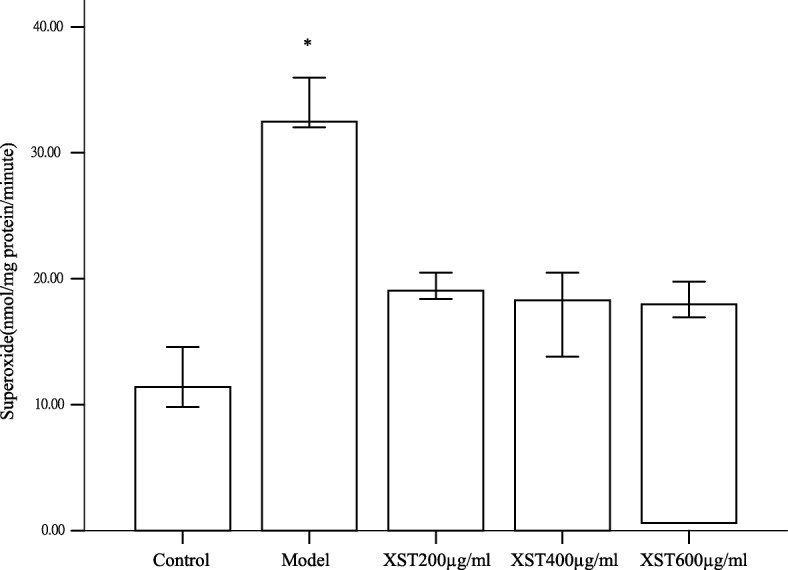
Mitochondrial ROS in the model group showed significantly higher than that in the control group, however, ROS levels exhibited a significant decrease in the XST-treated groups compared to those in the model group. No difference was noted between the three XST-treated groups. All samples were tested in three independent experiments with three replicates each. Data are represented as the mean ± SD. (^*^P<0.05 vs. each XST-treated group or the control group)

As shown in Fig. 7, the mitochondrial ROS in the model group was about three times higher than that in the control group (*P*<0.05), however, all the three XST-treated groups exhibited a significant decrease in ROS levels compared to the model group (*P*<0.05), no difference was noted between the three dosages of XST-treated groups (*P* > 0.05). Previous studies reported that the increase in mitochondrial Δψm and ATPase activity led to the decrease in mitochondrial ROS production [18]. Here, we confirmed that the XST-induced increase in Δψm and ATPase activity was coupled with a decrease in ROS. The data suggested that the three dosages of XST had the similar inhibitory effects on the production of mitochondrial ROS in hypoxia-reoxygenated HCM.

### XST decreased the apoptosis cells in the hypoxia-reoxygenated HCM

**Fig. 8:**
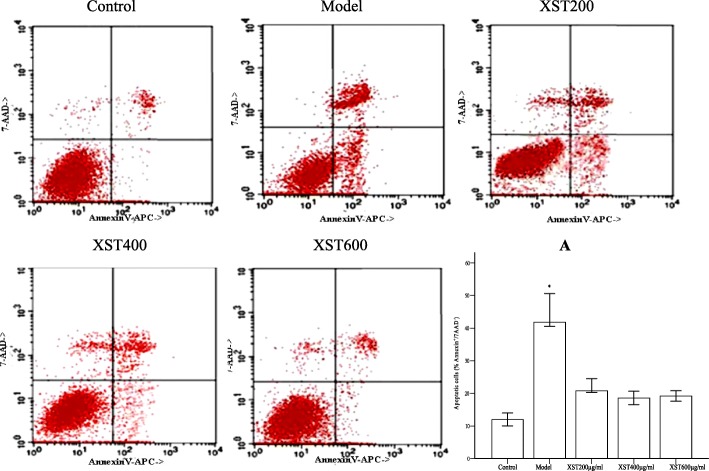
Five groups were subjected to an assessment of apoptosis cells in the typical diagrams by flow cytometry (annexinV^+^/7-AAD^−^). The percentage of annexinV^+^/7-AAD^−^, as the index of the apoptosis cells, increased in the model group. XST-treated groups declined the apoptosis cells as indicated by the quantitative bar graph (**a**). All samples were tested in three independent experiments with three replicates each. Data are represented as the mean ± SD (^*^P < 0.05 vs. the control or the XST-treated groups)

As detected by flow cytometry, apoptosis cells, which stained with annexinV+/7-AAD-, were significantly increased in the model group than those in the control group (*P* < 0.05) (Fig. 8). XST treatment could significantly decrease the apoptosis cells; however, no difference was noted between the three XST-treated groups (*P* > 0.05). The quantitative assessment of the apoptosis cells was indicated by bar graphs Fig. 8a.

## Discussion

Mitochondria, as the dominant source of heart ATP, represent approximately one-third of the mass of the heart and play a critical role in maintaining cellular function. The mitochondrion is very susceptible to damage mediated by ischemia or ischemia/reperfusion. The damaged mitochondria cause a depletion in ATP and a release of cytochrome c, which leads to activation of caspases and onset of apoptosis [19–21]. As such, maintaining mitochondrial homeostasis is critical to cell survival.

Current experiments showed that XST could maintain the activities of mitochondrial electron transport chain complexes I-IV, and restore the mitochondrial Δψm and mitochondria mass induced by oxidative stress in the cardiomyocytes. Further, the drug could activate mitochondrial total ATPase to supply cells energy to raise the cell viability in the anoxic conditions. On the other hand, XST could suppress the mitochondrial ROS generation and attenuate the ROS-induced damage to the cells, which may partly account for this drug’s inhibition of apoptosis and the increase in cell viability. The current data suggested that XST exerted its cardioprotective effects via maintaining the integrity of mitochondria in the hypoxia-reoxygenated HCM.

Our previous clinical study showed that the CHF patients treated with XST (0.3 g each capsule, three capsules each time, tid.) showed significant improvement in LVEF, NYHA classes, as well as the symptoms (dyspnea, edema and etc.) and the patients’ quality of life [22]. Further, in vitro experiments verified that dosages 200-600 μg/ml had the optimal cell protective effects in the oxidative stress conditions [11]. Based on the previous data, we used these dosages in the current study, which confirmed that XST could restore the ΔΨm and mitochondria mass to elevate the polarized cell populations. Treated at XST600μg/ml, the activities of respiratory chain complexes I-IV regained their activities nearly to the normal levels, and the total of mitochondrial ATPase activity was raised by XST. On the other hand, XST suppressed the generation of mitochondrial ROS and cell apoptosis. The data may partly shed a light on the drug’s therapeutic effects on the CHF patients.

Previous studies showed that an elevated ΔΨm was associated with the enhanced mitochondrial ROS formation [18, 23], and a slight decrease in ΔΨm could prevent ROS formation without seriously compromising cellular energetics [18, 24, 25]. However, in the isolated energized cardiac mitochondria, which were induced by hypoxia, caused the inhibition of the activity of the electron transport chain, and this mild decrease in ΔΨm led to ROS formation during reoxygenation [26]. The collapse of mitochondrial ΔΨm, regarded as an index of mitochondrial inner integrity, would go hand in hand with the dysfunctions of the mitochondrial respiratory chain and a decline of ATPase activity, which led to the elevation of mitochondrial ROS formation and cell apoptosis. The phenomena were supported in the current experiments as showed in Figs. 3, 5 and 7. XST treatment could restore the loss of ΔΨm and repaired the dysfunctions of the mitochondrial respiratory chain. Additionally, XST could activate ATPase and suppress ROS formation, resulted in the decline in apoptosis cells in the hypoxia-reoxygenated HCM. These data suggested that XST possibly exerted its cardioprotective effects partly through mitochondria. Mitochondrial respiratory chain, especially, complex I, III and III, were seen as the prime source of ROS [27–30]. ROS generations are decreased when the available electrons are limited and potential energy for the transfer is low [30].

Recent study found that when the electron transport chain functions of complexes I, III and III are at the sub optimal level, the rate of mitochondrial free radical production is inversely increasing proportional to the rate of electron transport [31], suggesting that the elevated ROS levels found under these conditions may originate extra-mitochondrially or are attributed to the defective antioxidant defense. Our experiments showed that XST-induced elevation of mitochondrial respiratory chain complexes activities was inversely correlated with the levels of mitochondria-derived ROS in the XST-treated groups (Figs. 5 and 7). Possibly, the drug suppressed ROS generation extra-mitochondrially, or through the antioxidant defense or other mechanisms. For example, other powerful sources of mitochondrial ROS production are represented by the mitochondrial NO synthase [32, 33], and the byproducts of several cellular enzymes including NADPH oxidases, xanthine oxidase [22, 23, 34]. Nevertheless, further studies should be taken to investigate the XST’s specific mechanisms on inhibition of ROS generation.

Impaired ΔΨm, a sign of the early stage of cell apoptosis [35], occurred before nucleus apoptosis characteristics (chromatin condensed and DNA rupture). Once the mitochondrial transmembrane potential collapse, apoptosis procedure is irreversible [36]. In this experiment, we confirmed that the decreased ΔΨm was associated with the increase in the apoptosis cells (Figs. 5 and 6). XST, via elevation of ΔΨm and inhibition of mitochondrial ROS generation, exerted its anti-apoptotic effects in the hypoxia-reoxygenated HCM. Conclusively, the drug, via ensuring the integrity of the mitochondrial membrane, exerted its cardioprotective effects in the hypoxia-reoxygenated HCM.

## Abbreviations

CHF: Chronic heart failure
ETC: Mitochondrial electron transport chain
HCM: Primary human cardiomyocyte
LVEF: Left ventricular ejection fraction
MPTP: Mitochondrial permeability transition pore
MTT: 3-[4,5-dimethylthiazol-2-yl]-diphenyl-tetrazolium bromide
NYHA: New York Heart Association
PBS: Phosphate-buffered saline
Pi: Inorganic phosphorus
ROS: Reactive oxygen species
SOD: Superoxide dismutase
XST: Xinshuitong Capsule
ΔΨm: Mitochondrial membrane potential
